# Biomechanical Effect of Distal Tibial Oblique Osteotomy: A Preliminary Finite-Element Analysis

**DOI:** 10.7759/cureus.53803

**Published:** 2024-02-07

**Authors:** Tatsuya Sakai, Masanori Fujii, Kenji Kitamura, Hirofumi Tanaka, Masaaki Mawatari

**Affiliations:** 1 Orthopedic Surgery, Saga University, Saga, JPN; 2 Orthopedic Surgery, Graduate School of Medical Sciences, Kyushu University, Fukuoka, JPN; 3 Orthopedic Surgery, Hyakutake Orthopedics and Sports Clinic, Saga, JPN

**Keywords:** joint contact area, finite element analysis, distal tibial oblique osteotomy, biomechanics, ankle osteoarthritis

## Abstract

Background: The biomechanical effect of distal tibial oblique osteotomy (DTOO) on osteoarthritic ankles has not been investigated. Using finite element (FE) models, we aimed to elucidate the effect of DTOO on the ankle contact pressure (CP) distribution.

Methods: This study included two patients with ankle osteoarthritis who underwent DTOO and one asymptomatic control. Patient-specific FE models were reconstructed by matching standing radiographs with supine computed tomography scans. The joint contact area (CA) and maximum CP on the articular surface of the talus were calculated before and after DTOO and compared with those of the control.

Results: In the control, the CA was 584 mm^2^ and the maximum CP was 2.6 MPa. In case 1, the CA increased by 125% from 166 mm^2^ preoperatively to 375 mm^2^ postoperatively, accompanied by a 36% decrease in the maximum CP from 9.8 MPa to 6.3 MPa. Similarly, in case 2, the CA increased by 46% from 301 mm^2^ to 439 mm^2^, accompanied by a 27% decrease in the maximum CP from 6.7 MPa to 4.9 MPa.

Conclusions: This study suggests DTOO improves the biomechanics of the ankle, but not sufficiently compared to the control. This analytical approach may enhance understanding of ankle pathophysiology and assist in the design of the ideal corrective osteotomy.

## Introduction

Ankle osteoarthritis is a condition characterized by progressive articular cartilage degeneration and reactive bone deformities, often associated with abnormal alignment, joint instability, and abnormal joint stress concentrations, which can predispose patients to significant disability and impair their quality of life [1-3]. The etiology and resulting deformities of ankle osteoarthritis vary widely. Adult flat feet tend to predispose patients to valgus osteoarthritis, while idiopathic osteoarthritis often manifests as varus osteoarthritis [1]. Distal tibial oblique osteotomy (DTOO) serves as a corrective osteotomy for varus osteoarthritis with the goal of stabilizing the tibiotalar joint, increasing the contact area (CA), and redistributing joint contact pressure (CP) to relieve pain and improve ankle function [4-6]. Despite encouraging clinical results [6-8], no study has validated the biomechanical improvements in the ankle after DTOO. Furthermore, there exists a lack of concrete evidence to direct the surgical indications for DTOO or to establish an association between morphological correction and treatment efficacy, leaving the decision largely at the discretion of the surgeon.

Finite element (FE) analysis has proven to be an effective tool for noninvasively simulating musculoskeletal biomechanics on a patient-specific basis. Previous FE models have effectively replicated the biomechanical environment of normal ankle joints, demonstrating nearly identical contact stress profiles between cadaveric and FE models [9]. Although some studies have used FE models to validate the therapeutic efficacy of artificial implants for the treatment of ankle osteoarthritis [10-12] to the best of our knowledge, no study has used these models to investigate the pathophysiology of ankle osteoarthritis or the efficacy of corrective osteotomy.

This study aimed to improve our understanding of the pathophysiology of ankle osteoarthritis and the potential benefits of corrective osteotomy by validating the biomechanical effects of DTOO on the tibiotalar joint using patient-specific FE analysis. We hypothesized that DTOO would result in a more balanced distribution of joint CP, potentially alleviating symptoms and slowing the progression of ankle osteoarthritis.

## Materials and methods

Patients

Two patients who underwent DTOO for ankle osteoarthritis between November 2019 and July 2021 were included in this study. Standing anteroposterior and mediolateral lower-leg radiographs and supine ankle computed tomography (CT) scans (matrix, 512 × 512; field of view, 250-700 mm; slice thickness, 1 mm) were obtained preoperatively and at the six-month follow-up. A board-certified orthopedic surgeon performed a medical record review to extract demographic data, including age, sex, and Japanese Society for Surgery of the Foot (JSSF) score [13]. Case 1 was a 73-year-old woman, and case 2 was a 65-year-old woman both with stage 3a osteoarthritis of the left ankle according to the Takakura-Tanaka classification [2,14].

As a control, the right ankle of a 53-year-old man with no history of ankle disease or symptoms and no osteoarthritis was included. Standing lower-leg radiographs and CT scans were obtained as preoperative examinations for high tibial osteotomy.

Morphological parameters were measured on the standing anteroposterior and mediolateral lower-leg radiographs, including tibial articular surface angle (TAS), medial malleolar angle (MMA), tibial lateral surface angle (TLS), talar tilt angle (TTA), and tibiotalar surface angle (TTS) [6,15].

Surgical procedure

The indication for DTOO was advanced to end-stage ankle osteoarthritis interfering with daily activities, i.e., stages 2-4 according to the Takakura-Tanaka classification [2,14] on the weight-bearing radiographs, with an ankle range of motion of 10° or greater [4-6]. The patients were placed in the supine position under general or spinal anesthesia. Dynamic instability of the tibiotalar joint was confirmed preoperatively. DTOO was performed through a skin incision on the medial side of the tibia, according to a previously described technique [4]. An oblique osteotomy was performed from the medial tibial cortex, approximately 5 cm proximal to the tibiotalar joint, to the distal tibiofibular joint. A laminar spreader was used to open the osteotomy site until the talus contacted the medial articular surface of the fibula and the tibiotalar joint was stabilized. Autologous or artificial bone was inserted into the osteotomy site and fixed with a locking plate. In cases with an ankle dorsiflexion angle of less than 0°, Achilles tendon lengthening was performed [6].

CT-based FE modeling

**Figure 1:**
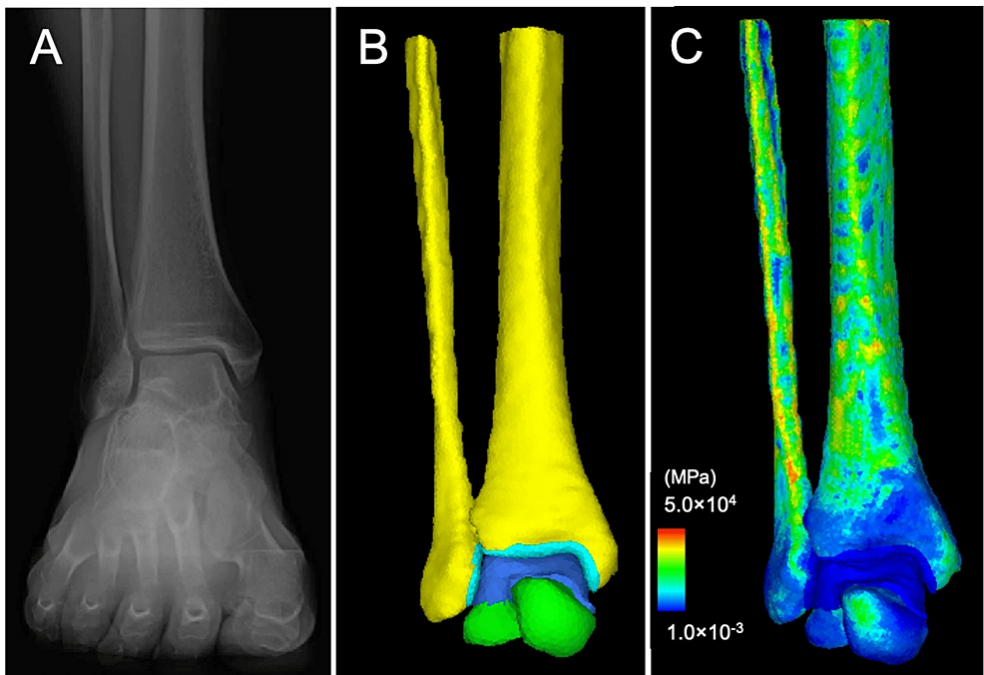
Control: Asymptomatic right ankle of a 53-year-old man. Anteroposterior weight-bearing radiograph of the ankle (A), the corresponding three-dimensional surface model, concretely, the tibia and fibula (yellow), talus (yellowish green), tibiofibular cartilage (light blue), and talar cartilage (blue) (B), and the corresponding finite element model with the distribution of the elastic modulus in a subject (C)

**Figure 2:**
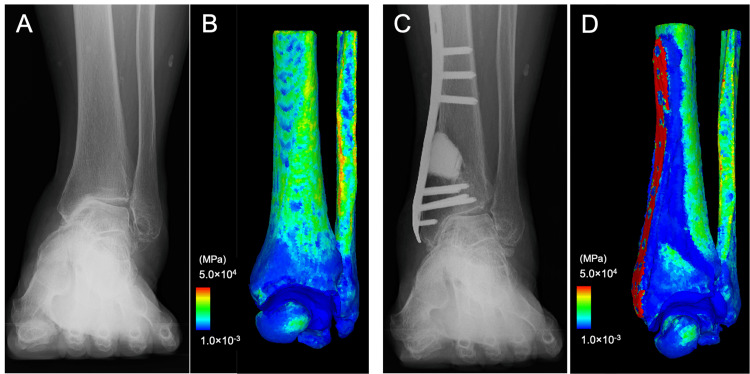
Case 1: A 73-year-old woman with stage 3a osteoarthritis of the left ankle. (A) preoperative anteroposterior weight-bearing radiograph of the ankle (B) the corresponding finite-element model with the distribution of the elastic modulus (C) postoperative anteroposterior weight-bearing radiograph of the ankle, and (D) the corresponding finite-element model with the distribution of the elastic modulus at six months after distal tibial oblique osteotomy

**Figure 3:**
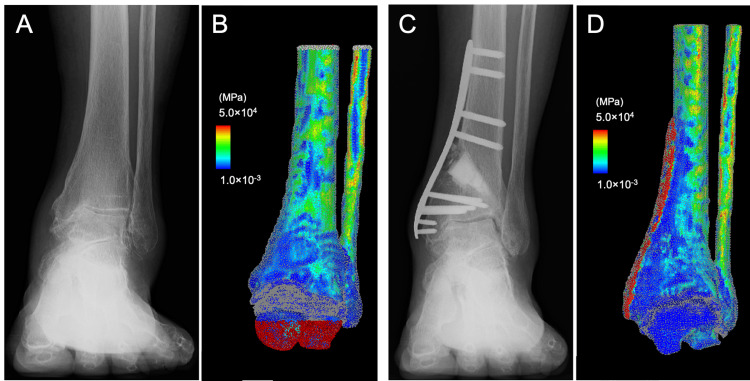
Case 2: A 65-year-old woman with stage 3a osteoarthritis of the left ankle. (A) preoperative anteroposterior weight-bearing radiograph of the ankle (B) the corresponding finite-element model with distribution of the elastic modulus (C) postoperative anteroposterior weight-bearing radiograph of the ankle, and (D) the corresponding finite-element model with the distribution of the elastic modulus at six months after distal tibial oblique osteotomy

Mechanical Finder version 10 (Computational Mechanics Research Center, Tokyo, Japan) was used to develop linear FE models describing the shape and density distribution of each bone visible on the CT scans. The Hounsfield unit (HU) threshold for bone model segmentation was set to 300 [16], with manual adjustment required when this value was <300. We constructed a three-dimensional FE model of the tibia and talus for each patient using previously established methods for the hip joint (Figures 1, 2, 3) [17,18]. Tibiotalar alignment in the standing position was reproduced by matching the morphology of the tibia and talus on standing anteroposterior and mediolateral lower-leg radiographs with digitally reconstructed coronal and sagittal radiographs. The trabecular and inner cortical bones were modeled using 2-mm tetrahedral elements, whereas the outer surface of the cortical bone was modeled using 0.4-mm-thick three-nodal point shell elements. The surface elements were selected to ensure that the models reflected the material properties of the bone, minimizing the impact of the partial volume effect owing to the very small CT values [19]. The cartilages of both the tibia and talus were modeled with a constant thickness of 1.7 mm [9]. The articular surface was discretized using tetrahedral elements with a thickness of 0.5-2.0 mm, with local refinement in the weight-bearing area of the articular cartilage. Three-nodal point shell elements with a thickness of 0.0005 mm were placed on the surface of the articular cartilage to visualize the CP on the cartilage. The articular cartilage was modeled as a homogeneous and isotropic material. The average quantities of FEs and shell elements were approximately 865,000 and 50,000 for the osteoarthritis model, and 870,000 and 47,000 for the control model, respectively. To account for bone heterogeneity, the distribution of bone mineral density (ρ in g/cm^3^) of each model component was calculated using a standardized equation for determining bone mineral density from HU as follows: ρ (g/cm^3^) = (HU + 1.4246) × (0.001/1.058 (HU value > -1), ρ(g/cm^3^) = 0 (HU value ≤ -1) [20]. Young’s modulus was determined from the bone density values using the equation of Keyak et al. [21], and Poisson's ratio of the bone, artificial bone, and implant was set to 0.3. The Young’s modulus and Poisson's ratio of the articular cartilage were set to 12 MPa and 0.42, respectively [9].

Boundary and loading conditions

FE models were employed to conduct a nonlinear contact analysis to calculate the joint CA and CP on the articular cartilage of the talus. Tied and sliding contact constraints were set on the cartilage-bone and cartilage-cartilage interfaces, respectively. The friction coefficient between the articular cartilage surfaces is reportedly very low (0.01-0.02) in the presence of synovial fluid, suggesting that it is reasonable to disregard frictional shear stresses [22]. The distal talus was fixed and the proximal tibia was constrained along the x and y axes, allowing unrestricted movement in the z-direction (vertical). The loading scenario was based on a single-leg stance, with the contact force acting on the tibiotalar joint surface of the talus. A consistent weight of 600 N was deﬁned for all patients to avoid the scaling effect of weight on absolute CP values, and a body weight equivalent load was applied to the proximal tibia along the z-axis [9]. For the nonlinear contact analysis, incremental loading was performed in 40 steps. The CA and CP at the articular surface of the talus before and after DTOO were calculated and compared with those of the control. No statistical analysis was performed on the comparison.

Ethics statement

Each author certifies that his institution approved the human protocol for this investigation and that all investigations were conducted in conformity with the ethical principles of research. Ethical approval for this retrospective study was obtained from the Faculty of Medicine, Saga University (2022-06-R-06). All data were fully anonymized before we accessed them, and all participants provided written informed consent to participate in this study.

## Results

Control

**Figure 4:**
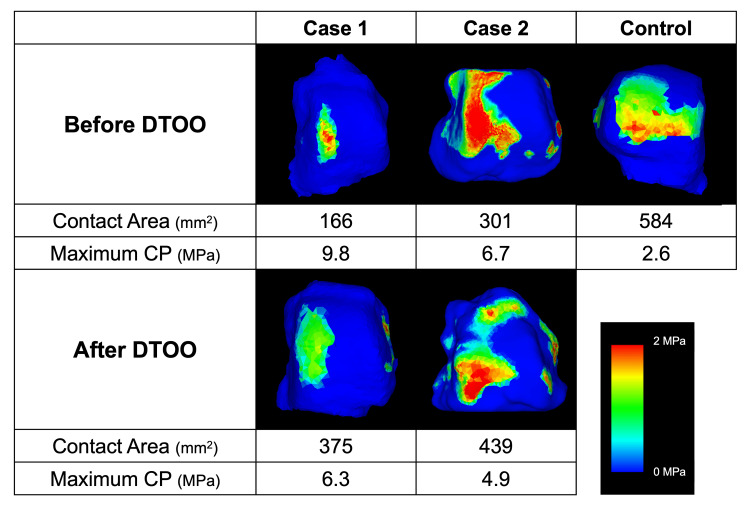
Distribution of joint contact pressure at the articular surface of the control and two patients before and after distal tibial oblique osteotomy DTOO: Distal tibial oblique osteotomy; CP: Contact pressure

The morphological parameters were TAS 90°, MMA 24°, TLS 84°, TTA 0°, and TTS 90° (Table 1). The JSSF score was 100. FE analysis (Figure 1) showed a CA of 584 mm² and a maximum CP of 2.6 MPa at the articular cartilage surface of the talus (Figure 4). These values served as baselines for evaluating biomechanical changes in the two cases after DTOO.

**Table 1:** Clinical and radiographic parameters TAS: Tibial articular surface angle; MMA: Medial malleolar angle; TLS: Tibial lateral surface angle; TTA: Talar tilt angle; TTS: Tibiotalar surface angle; JSSF: Japanese Society for Surgery of the Foot

		Clinical score	Radiographic parameters				
		JSSF score	TAS (°)	MMA (°)	TLS (°)	TTA (°)	TTS (°)
Control		100	90	24	84	0	90
Case 1	Preoperative	55	85	36	83	14	73
	Postoperative	97	106	26	85	13	91
Case 2	Preoperative	44	89	48	87	10	76
	Postoperative	85	102	26	78	4	96

Case 1

Morphological parameters improved after DTOO, with TAS changing from 85° to 106°, MMA from 36°to 26°, and TTS from 73° to 91°. However, TTA exhibited only minimal improvement (Table 1). The JSSF score improved from 55 preoperatively to 97 at the six-month follow-up. FE analysis (Figure 2) showed that the CA was limited to the medial side of the tibiotalar joint preoperatively, measuring 166 mm^2^. Postoperatively, the CA increased by 125% to 375 mm^2^ and the maximum CP decreased by 36% from 9.8 MPa to 6.3 MPa, indicating a more even redistribution of load across the tibiotalar joint (Figure 4).

Case 2

Morphological parameters improved after DTOO, with TAS changing from 89° to 102°, MMA from 48°to 26°, TTS from 76° to 96°, and TTA from 10° to 4° (Table 1). The JSSF score improved from 44 preoperatively to 85 at the six-month follow-up. Similar to the findings in case 1, the FE analysis (Figure 3) in case 2 showed a 46% increase in CA from 301 mm^2^ to 439 mm^2^. Concurrently, the maximum CP decreased by 27% from 6.7 MPa to 4.9 MPa, indicating a more uniform load redistribution across the tibiotalar joint (Figure 4).

Both cases 1 and 2 showed consistent increases in CA and maximum CP, supporting the potential biomechanical benefits of DTOO. However, the CP was still lower, and the maximum CP was higher after DTOO than in the control.

## Discussion

DTOO is a corrective osteotomy for ankle osteoarthritis designed to stabilize the tibiotalar joint, increase CA, and redistribute joint CP to relieve pain and improve ankle function [4-6]. Although clinical studies have shown promising results [6-8], biomechanical evidence supporting the efficacy of DTOO is lacking. Using patient-specific FE models, the current study demonstrated for the first time that DTOO effectively improved the biomechanics of ankle osteoarthritis, as indicated by increased CA and redistribution of CP. However, our results also suggested that this effect was not sufficient to normalize the biomechanical environment of the tibiotalar joint.

Ankle osteoarthritis is a common degenerative joint disorder characterized by articular cartilage degeneration and associated bone deformities, resulting in significant disability and impaired quality of life [1-3]. This disorder has a multifactorial etiology, including age-related reduction in cartilage viability, physical damage from acute stresses such as trauma, alignment abnormalities, chronic stresses from joint instability, and inflammatory diseases [23]. Secondary conditions account for >70% of cases [1], however, primary osteoarthritis, a predisposing factor for valgus osteoarthritis, is relatively common in Japan. This may be due to inherent abnormalities in the distal tibial articular surface combined with joint instability [24,25]. Distal tibial osteotomies are indicated for primary varus osteoarthritis depending on the severity of the osteoarthritis, with the goal of realigning and stabilizing the tibiotalar joint to relieve pain while maintaining the ankle range of motion [2,4]. Typically, a low tibial osteotomy is indicated for moderate-to-advanced cases (stages 2-3a) [2,14] whereas DTOO is indicated for more severe cases (stages 3b-4) [4]. However, comprehensive biomechanical validation of these osteotomies is lacking, resulting in a dearth of evidence-based guidelines for surgical indications and a paucity of data elucidating the relationship between morphological correction and therapeutic outcomes, leaving the decision largely at the surgeon’s discretion.

Although previous studies have used FE models to validate the therapeutic efficacy of artificial implants for ankle osteoarthritis [10-12] none have used FE models to investigate the pathophysiology of ankle osteoarthritis or the therapeutic efficacy of corrective osteotomy. Anderson et al. [9] demonstrated the validity of FE analysis of a normal ankle by direct comparative evaluation using a cadaver model. Although previous studies have used different loading conditions [10,26], the CA and maximum CP observed in the normal ankle in this study were consistent with those reported by Anderson et al. [9] under the same conditions; the CA ranged from 290.5 mm^2^ to 419.9 mm^2^ and the maximum CP ranged from 2.74 MPa to 3.74 MPa. Previous studies have employed FE analyses of the ankle joint [27,28]; however, most of these studies have focused on variations in CP during the gait cycle in healthy individuals [26,29] or on component loading after joint replacement [10-12]. In this study, we used patient-specific FE models to demonstrate that DTOO effectively improves the biomechanics of ankle osteoarthritis as evidenced by increased CA and redistribution of CP. These biomechanical changes were congruent with the postoperative improvement in clinical scores, highlighting that improvement in joint load distribution may contribute to pain reduction and improved functional outcomes. This FE modeling strategy has the potential to elucidate the biomechanical changes induced by DTOO, refine surgical planning, and establish appropriate expectations for clinical improvement after DTOO.

However, our results also indicated that the effect of DTOO was not sufficient to normalize the biomechanical environment of the tibiotalar joint; the postoperative maximum CP of 6.3 MPa in case 1 and 4.9 MPa in case 2, were still high compared with the corresponding control value. A plausible explanation for this denormalization is the irreversible articular cartilage degeneration at the time of surgery. Alternatively, the inherent procedural limitations of DTOO or specific deformity may preclude comprehensive biomechanical restoration. Regarding morphological improvement with DTOO, Harada et al. [5,6] demonstrated significant improvements in all the morphological parameters evaluated in this study, whereas Ahn et al. [8] found that the TTA levels did not substantially decrease after DTOO. This suggests that the tolerable preoperative limits for TTA in DTOO may be more stringent than those for low tibial osteotomy. In this study, both biomechanical parameters and clinical scores improved after DTOO in both cases; however, case 1 showed a decreased CA and increased maximum CP compared to case 2, in which TTA showed improvement. Although these results suggest an association between morphological correction by DTOO and changes in the biomechanical environment, a more extensive longitudinal study with a larger cohort of patients is required to holistically understand the association between the morphological and biomechanical changes induced by DTOO and their subsequent clinical implications.

There are several limitations to this study. First, this was a preliminary study with only two patients undergoing DTOO, thereby limiting the generalizability of our findings. Furthermore, the six-month follow-up period may be insufficient to fully elucidate the long-term biomechanical effects and clinical efficacy of DTOO. Nevertheless, our preliminary data provide valuable insights into the biomechanical effects of DTOO and serve as a critical foundation for future research on this topic. Additional longitudinal studies with larger cohorts are required for a more comprehensive understanding of the biomechanical and clinical effects of DTOO. Second, the biomechanical assessment was limited to a single loading scenario representing a single-leg stance, using simplified loading conditions with fixed x and y axes. This approach did not include an assessment of stress distribution under alternative loading conditions relevant to daily activities and the gait cycle [29,30]. Additionally, simplified modeling without the entire foot alignment, such as the calcaneus and other tarsals, may influence our observations. Therefore, the practical applicability of our conclusions may be limited. Future studies should include models representing the alignment and biomechanics of the entire foot, as well as a range of loading conditions to provide a more comprehensive perspective on the biomechanical consequences of DTOO.

## Conclusions

This FE model study suggests that DTOO effectively improves the biomechanics of ankle osteoarthritis, which is evident in increased CA and redistribution of CP; however, the outcome was not sufficient to normalize the biomechanical environment. This analytical approach holds promise for enhancing our understanding of ankle osteoarthritis pathophysiology, elucidating the impact of corrective osteotomy on ankle biomechanics, refining surgical planning, and establishing realistic expectations for clinical improvement after corrective osteotomy. Future studies should explore factors influencing biomechanical improvement, the degree of adequate correction, and their relationship with clinical outcomes.
